# Spatiotemporal pattern of hemorrhagic fever with renal syndrome and driving factors in Shandong Province of China, 2018–2024

**DOI:** 10.1371/journal.pntd.0014023

**Published:** 2026-02-24

**Authors:** Qing Duan, Yinlong Li, Suying Guo, Shiyi Huang, Yan Li, Yuwei Zhang, Ruixiao Li, Shan Lv, Shizhu Li, Jing Xu, Zengqiang Kou, Ti Liu

**Affiliations:** 1 National Institute of Parasitic Diseases, Chinese Center for Disease Control and Prevention, Chinese Center for Tropical Diseases Research, NHC Key Laboratory of Parasite and Vector Biology, WHO Collaborating Centre for Tropical Diseases, National Center for International Research on Tropical Diseases, Ministry of Science and Technology, Shanghai, China; 2 Shandong Provincial Center for Disease Control and Prevention, Jinan, China; 3 Shandong Provincial Key Laboratory of Intelligent Monitoring, Early Warning, Prevention and Control for Infectious Diseases, Jinan, China; 4 School of Global Health, Chinese Center for Tropical Diseases Research, Shanghai Jiao Tong University School of Medicine, Shanghai, China; Asahikawa Medical University: Asahikawa Ika Daigaku, JAPAN

## Abstract

**Background:**

Hemorrhagic fever with renal syndrome (HFRS) is a widespread zoonotic disease transmitted by rodents, posing a serious public health threat in People’s Republic of China. Due to the higher incidence of HFRS occurred in Shandong Province, this study aims to understand the spatiotemporal pattern of HFRS, identify the driving factors and predict potential high-risk areas in Shandong Province, to provide guidance for public health policy making.

**Methods:**

Case information on HFRS occurred in Shandong Province from 2018 to 2024 was collected from the China Information System for Disease Control and Prevention (CISDCP). Incidence rate of HFRS was calculated monthly and annually to explore its preliminary distribution trend. Spatiotemporal scanning analysis was used to determine the temporal and spatial clustering characteristics of HFRS cases. The maximum entropy (MaxEnt) model was employed to explore the major factors influencing HFRS and predict high-risk areas of HFRS in Shandong Province.

**Results:**

From 2018 to 2024, a total of 4,837 cases of HFRS were reported in Shandong Province, with the incidence rate showing a fluctuating downward trend. The peak incidence period occurred annually from October to December. Spatiotemporal scanning analysis showed the first cluster involved 29 counties across 5 prefecture-level cities in eastern Shandong Province, spanning October to November 2018. The second cluster involved 16 counties across 6 prefecture-level cities in central Shandong Province, spanning November to December 2021. The third cluster area involved 4 counties across 2 prefecture-level cities in southwestern Shandong Province, spanning March to April 2018. The fourth cluster area was located in Shanghe County, north of Jinan City, spanning November to December 2021. When optimizing the MaxEnt model, the optimal performance was achieved with the feature class (FC) set to linear, quadratic, hinge, product, and threshold (LQHPT) and the regularization multiplier (RM) set to 0.2. Yearly average air temperature, normalized difference vegetation index, yearly average relative humidity and yearly average sunshine duration were identified as the main factors influencing the occurrence of HFRS. The risk prediction map showed that high-risk areas for HFRS were primarily concentrated in the eastern and central regions of Shandong Province, covering an area of 26682.92 square kilometers, accounting for 16.90% of the province’s total area.

**Conclusion:**

HFRS in Shandong Province exhibited obvious spatiotemporal patterns and was influenced by multiple factors, including temperature, vegetation, humidity and sunshine. These findings highlight the need for health authorities to integrate environmental and socio-economic considerations into the design of strategy or countermeasures against HFRS, particularly during high-incidence season and in high-risk areas.

## Author summary

Shandong Province is a historical HFRS endemic area in China. However, recent epidemiological studies, particularly those investigating its transmission patterns, have been limited. Utilizing spatiotemporal scan analysis and the MaxEnt model, this study elucidated the distribution pattern, identified key drivers, and predicted high-risk areas for HFRS in Shandong Province (2018–2024). The study revealed significant spatiotemporal patterns of HFRS existed in Shandong Province. The results indicated that HFRS incidence was driven by multiple factors, with temperature, vegetation, humidity and sunshine being the key variables. The coastal areas of eastern Shandong and the inland regions of central Shandong were high-risk areas for HFRS. This study provides valuable insights for public health authorities to develop scientifically sound and reasonable HFRS prevention and control strategies, and also serves as a reference for epidemic prevention and control in similar HFRS-endemic regions.

## Introduction

Hemorrhagic fever with renal syndrome (HFRS) is a widespread zoonotic disease caused by the rodent-transmitted orthohantaviruses [1]. Inhalation of aerosols formed from the saliva, urine, feces, and other excretions of rodents is the most common mode of transmission [2–4]. HFRS has an acute onset and rapid progression, with diverse clinical features, but the most typical symptoms include fever, bleeding, hypotension, headache, abdominal pain, and acute renal failure, with a case-fatality rate as high as 15% [5–7]. It primarily circulates in Eurasia, with annual global case reports ranging from 60,000–150,000, involving over 70 countries [1,8]. Although the disease has been reported in multiple countries, there are significant variations in prevalence across different regions. These differences can be attributed to various factors, including variations in rodent populations, climatic conditions, and human behavior. China is the country with the highest number of reported HFRS cases globally, accounting for over 90% of all reported cases worldwide annually. From 1995 to 2020, the cumulative number of reported cases exceeded 570,000 [9,10]. As the most affected country, China has reported HFRS cases in all 31 provinces (autonomous regions and municipalities) [11]. Shandong Province identified its first case of HFRS as early as 1968. It is a historically endemic area for HFRS and one of the provinces with the highest incidence rates in China.

A notable feature of HFRS is the close evolutionary association between orthohantaviruses and certain rodent species, with each orthohantavirus having its specific host [12]. There are many rodent species capable of carrying orthohantaviruses, with the *Rattus norvegicus* and *Apodemus agrarius* being the most common in China [13]. *Rattus norvegicus* frequently carry Seoul virus (SEOV), with a peak incidence in spring. *Apodemus agrarius* commonly carry Hantaan virus (HTNV), with a peak incidence in autumn and winter. In regions where both rodent species coexist, two epidemic peaks occur annually [14]. Each epidemic region typically remains stable over a certain period, but the prevalence of HFRS is still influenced by multifaceted environmental factors, including natural, social, and human factors. Previous studies have shown that meteorological factors such as temperature and precipitation are closely associated with the distribution of HFRS [2,15,16], while human and social factors such as population density, housing type, and the extent of human contact with epidemic foci are also linked to the transmission of HFRS [9,17].

In China, previous studies on HFRS have primarily focused on epidemiological characteristics, time-series characteristics, and spatial clustering. However, research on the pathogenesis of HFRS at both temporal and spatial scales, particularly at the level of fine-grained spatial units, remains limited. The Maximum Entropy (MaxEnt) model is a type of ecological niche model that is based on the relationship between a species and its surrounding environment. It was originally used to understand the ecological requirements of species and predict specific suitable habitats [18,19]. However, in recent years, MaxEnt has been widely applied in the field of infectious disease research to analyze the influencing factors of disease occurrence at the fine-scale spatial unit level and predict potential risk areas [20]. Meanwhile, MaxEnt itself has continued to evolve, with an increasing number of scholars using optimized MaxEnt for species distribution modeling. This method significantly improves the precision of data analysis; however, it has not yet been applied to HFRS on a global scale. This study describes the spatiotemporal distribution of HFRS in Shandong Province, China, and for the first time uses optimized MaxEnt to analyze influencing factors and predict high-risk areas, providing scientific basis for developing targeted HFRS prevention and control strategies.

## Materials and methods

### Research area

Shandong Province (34°22.9′N - 38°24.01′N, 114°47.5′E - 122°42.3′E) is located on the eastern coast of China, downstream of the Yellow River, bordering the Yellow and Bohai Seas to the east, and sharing borders with Hebei, Henan, Anhui, and Jiangsu provinces inland (S1 Fig). The climate is a typical warm temperate monsoon climate, with distinct seasons. Summers are hot and rainy, while winters are cold and dry. The terrain is primarily plains and hills, with Mount Tai rising prominently in the central region, plains in the southwest and northwest, and the Shandong Peninsula hills in the east. Shandong administers 16 prefecture-level cities and 136 counties. As a province with a permanent population exceeding 100 million, it ranks second in the country in terms of population size [21].

### Data collection

HFRS case data are sourced from the infectious disease surveillance module of the China Information System for Disease Control and Prevention (CISDCP). After anonymization, the collected information includes age, gender, occupation, case type, diagnosis date, and current residential address. Annual resident population data for each region are sourced from the basic information module of the CISDCP system.

Meteorological data were obtained from the China Meteorological Data Sharing Service System (http://data.cma.cn/). Topographic data and socioeconomic data were collected from the Resource and Environment Science and Data Center (http://www.resdc.cn/) and the Geospatial Digital Cloud Platform (http://www.gscloud.cn/) (Table 1). All variables were uniformly processed into ASCII raster format using ArcGIS 10.7 software, with a spatial resolution of 1 km × 1 km.

**Table 1:** Explanation of environmental variables and their contribution to the MaxEnt model.

Category	Abbreviation	Variable	Whether excluding	Percentage contribution (%)
Meteorological data	EVP	Yearly average evaporation (mm)	Yes	–
	GST	Yearly average ground surface temperature (°C)	No	4.8
	PRE	Yearly average precipitation (mm)	Yes	–
	PRS	Yearly average atmospheric pressure (hPa)	No	3.3
	RHU	Yearly average relative humidity (%)	No	12.2
	SSD	Yearly average sunshine duration (h)	No	11.2
	TEM	Yearly average air temperature (°C)	No	19.9
	WIN	Yearly average wind speed (m/s)	No	8.3
Topographical data	ELE	Elevation (m)	No	4.9
	ASP	Aspect (rad)	Yes	–
	SLO	Slope (°)	No	7.5
	NDVI	Yearly normalized difference vegetation index	No	19.2
Socioeconomic data	GDP	Cross domestic product (10^4^ RMB/person)	Yes	–
	NPP	Nighttime light index	No	8.8
	HFP	Human footprint index	Yes	–
	POP	(persons/km^2^)	Yes	–

### Spatiotemporal analysis

The spatiotemporal clustering of HFRS was assessed using the SatScan 10.0.1 software. SatScan is an open-source software based on the fundamental principles of scan statistics, which can be categorized into pure temporal scans, pure spatial scans, and spatiotemporal scans depending on the scale of the scan [22]. This study selected the spatiotemporal scan for analysis because it can simultaneously examine characteristics in both the temporal and spatial dimensions. The scan window is cylindrical, with the base of the cylinder representing the spatial dimension and the height of the cylinder representing the temporal dimension. The theoretical number of cases was calculated using a discrete Poisson distribution model, and a log-likelihood ratio (LLR) was constructed using the actual number of cases. The cluster with the maximum LLR is considered the most probable cluster, meaning the cluster least likely to be caused by chance.

In practice, counties are used as the basic geographical units and months as the basic time units. The maximum risk population is set at 25% of the total population, and the maximum time scan window is set at two months. The Monte Carlo simulation method is used to calculate the P-value in the LLR test, with 999 simulations. Rank the clusters in order according to the size of the LLR. ArcGIS 10.7 software is used for visualization.

### The MaxEnt model

To identify potential environmental risk factors, quantify their interaction with HFRS occurrence, and create a risk prediction map, an ecological niche model was constructed using the maximum entropy algorithm. MaxEnt is an artificial intelligence model based on machine learning technology and is currently widely used for assessing the factors influencing infectious diseases and spatial prediction [23,24]. To avoid multicollinearity issues, specific values for each environmental variable were extracted using ArcGIS 10.7 software prior to modeling. Principal component analysis was then performed using R 4.5.1 software to screen the variables. When performing principal component analysis, the 16 variables were first standardized. The loadings and overall contribution of each variable on the principal components were calculated. The screening principle prioritized representative variables with high loadings across different principal components. The top 10 key variables were selected based on their overall contribution ranking. This method effectively reduces multicollinearity while retaining most environmental information, ensuring the stability and interpretability of the subsequent MaxEnt model. Additionally, considering that HFRS distribution data may be influenced by diagnostic differences, leading to overly dense disease distribution in specific regions and potentially causing overfitting in the research results, data screening was conducted using ENMTools 1.4 software to ensure that only one point was retained per 1 km × 1 km grid in the environmental raster data.

During the modeling process, considering that the predictive performance of the model may be affected by the regularization multiplier (FC) and feature combination (RM), the “ENMeval” package in R 4.3.1 software was used for parameter optimization. Six FC values (L, H, LQ, LQH, LQHP, LQHPT) and 20 RM levels (ranging from 0.2 to 0.4, with intervals of 0.2) were combined, and the Akaike Information Criterion (AICc) was calculated to assess model fit and complexity. The combination with the minimum AICc value (DAICc = 0) was selected for inclusion in the model to enhance its predictive performance.

Additionally, 75% of the data was randomly selected as the training set, and 25% as the test set. The Bootstrap method was used, with the default maximum background point count set to 10,000, and a random seed was enabled to ensure reproducible results. The number of repetitions is set to 10, and the output results are set to logical values. The relationship between each variable and HFRS occurrence is evaluated by plotting response curves, and the relative importance of different variables is assessed using the jackknife analysis. Variables with a contribution greater than 10% are considered to be highly correlated with HFRS occurrence.

AUC refers to the area under the ROC (receiver operating characteristic) curve, which is commonly used to assess model accuracy and is not influenced by the proportion of subjects in the sample. In this study, AUC was used to evaluate model performance. An AUC value <0.7 indicates low model accuracy, 0.7–0.9 indicates moderate accuracy, and >0.9 indicates high accuracy [20]. The risk prediction map was divided into non-prevalence, low-prevalence, mid-prevalence, and high-prevalence areas using the natural breakpoint method. Modeling was performed using MaxEnt 3.4.1 software, and the results were visualized using ArcGIS 10.7 software.

## Results

### Descriptive analysis of HFRS cases

**Fig 1:**
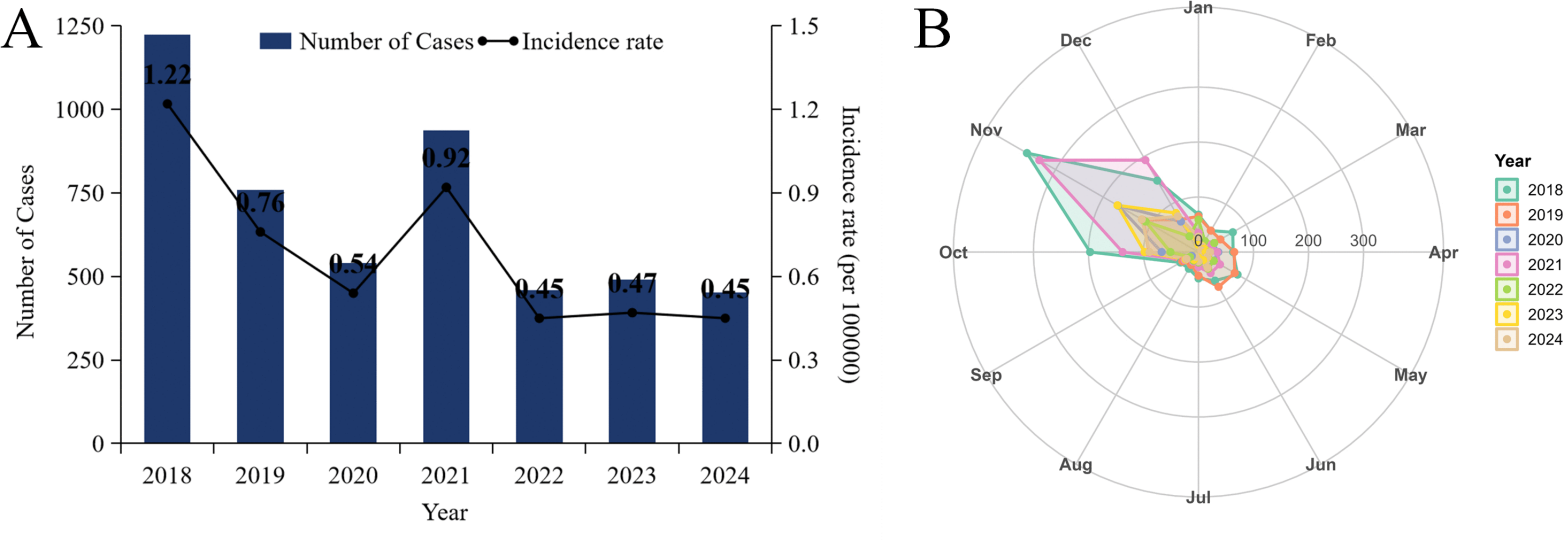
General characteristics of HFRS in Shandong Province from 2018 to 2024. **(A)** Number of reported cases and incidence rate of HFRS per year; **(B)** Monthly reported number of HFRS cases.

From 2018 to 2024, Shandong Province reported a cumulative total of 4,837 cases of HFRS, with 71 deaths, resulting in a case-fatality rate of 1.47%. The incidence rate showed a fluctuating downward trend, decreasing from 1.22 per 100,000 in 2018 to 0.45 per 100,000 in 2024, with an average annual incidence rate of 0.69 per 100,000. By gender, there were 3,541 male cases and 1,296 female cases, with a male-to-female ratio of 2.73:1. By age, the majority of cases were in middle-aged and older adults aged 40 and above, accounting for 81.2%. By occupation, the majority were farmers, workers, and unemployed individuals, accounting for 79.2%, 5.4% and 5.3%, respectively. From a temporal distribution perspective, the peak incidence period occurred annually from October to December, accounting for 57.4% of all reported cases (Fig 1 and S1 Table).

### Spatiotemporal clusters of HFRS

**Fig 2:**
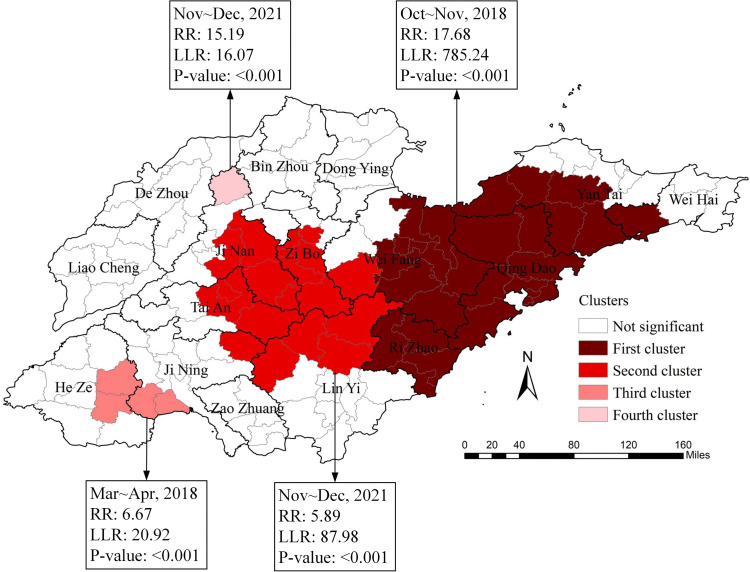
Spatiotemporal clusters of HFRS in Shandong Province from 2018 to 2024. The base map is from the National Platform for Common GeoSpatial Information Services (https://cloudcenter.tianditu.gov.cn/administrativeDivision).

From 2018 to 2024, the distribution of HFRS in Shandong Province exhibited four spatiotemporal clusters. The first cluster was located in eastern Shandong Province, encompassing 29 counties across 5 prefecture-level cities, with a time span from October to November 2018 (RR = 17.68, LLR = 785.24, P < 0.01). The second cluster was located in central Shandong Province, encompassing 16 counties across 6 prefecture-level cities, with a time span from November to December 2021 (RR = 15.19, LLR = 16.07, P < 0.01). The third cluster was located in southwestern Shandong Province, encompassing 4 counties across 2 prefecture-level cities, with a time span from March to April 2018 (RR = 6.67, LLR = 20.92, P < 0.01). The fourth cluster was located in Shanghe County, north of Jinan City, with a time span from November to December 2021 (RR = 15.19, LLR = 16.07, P < 0.01) (Fig 2 and S2 Table).

### Driving factors and high-risk regions revealed by the MaxEnt model

**Fig 3:**
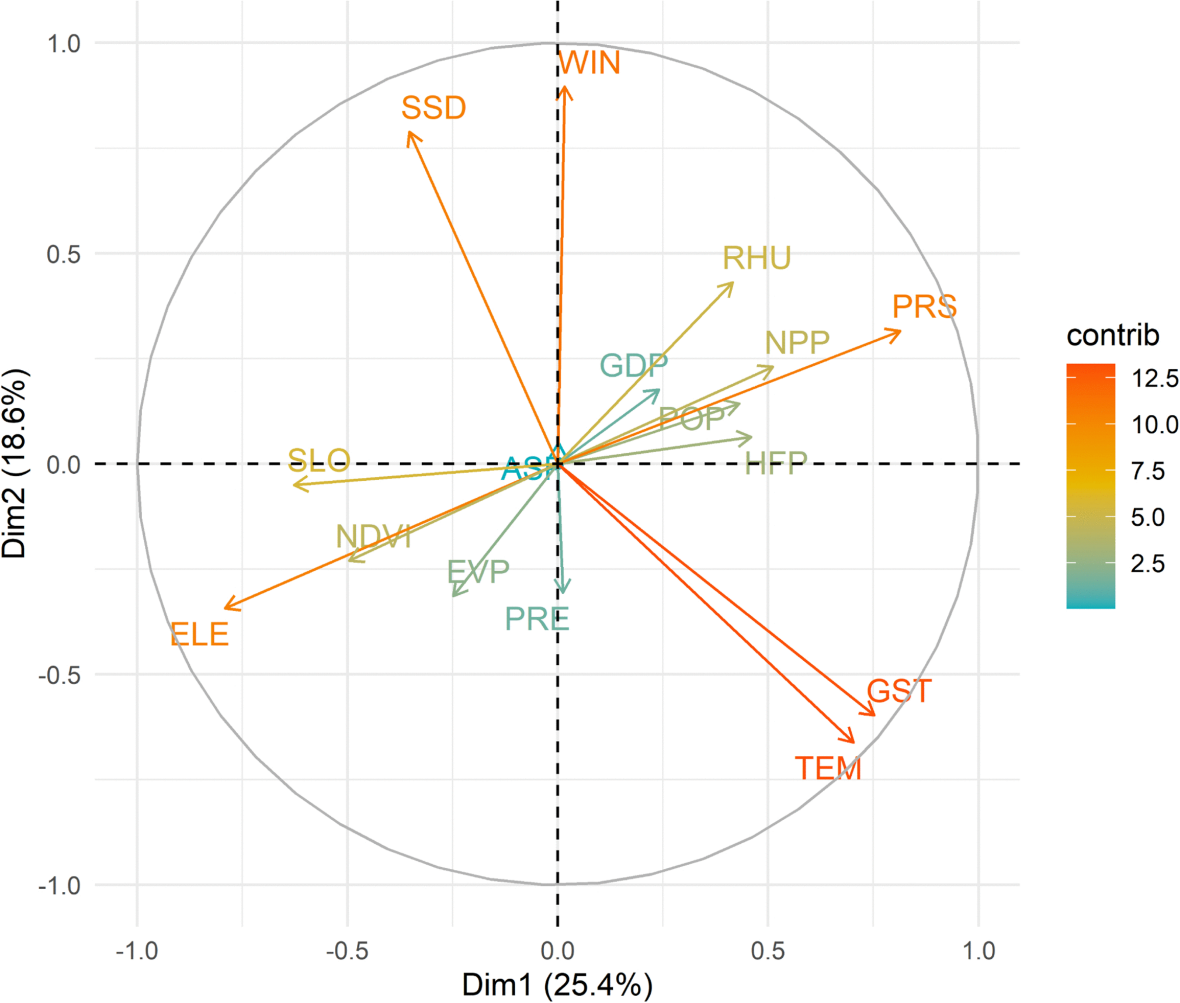
Distribution of variables in the principal component space.

**Fig 4:**
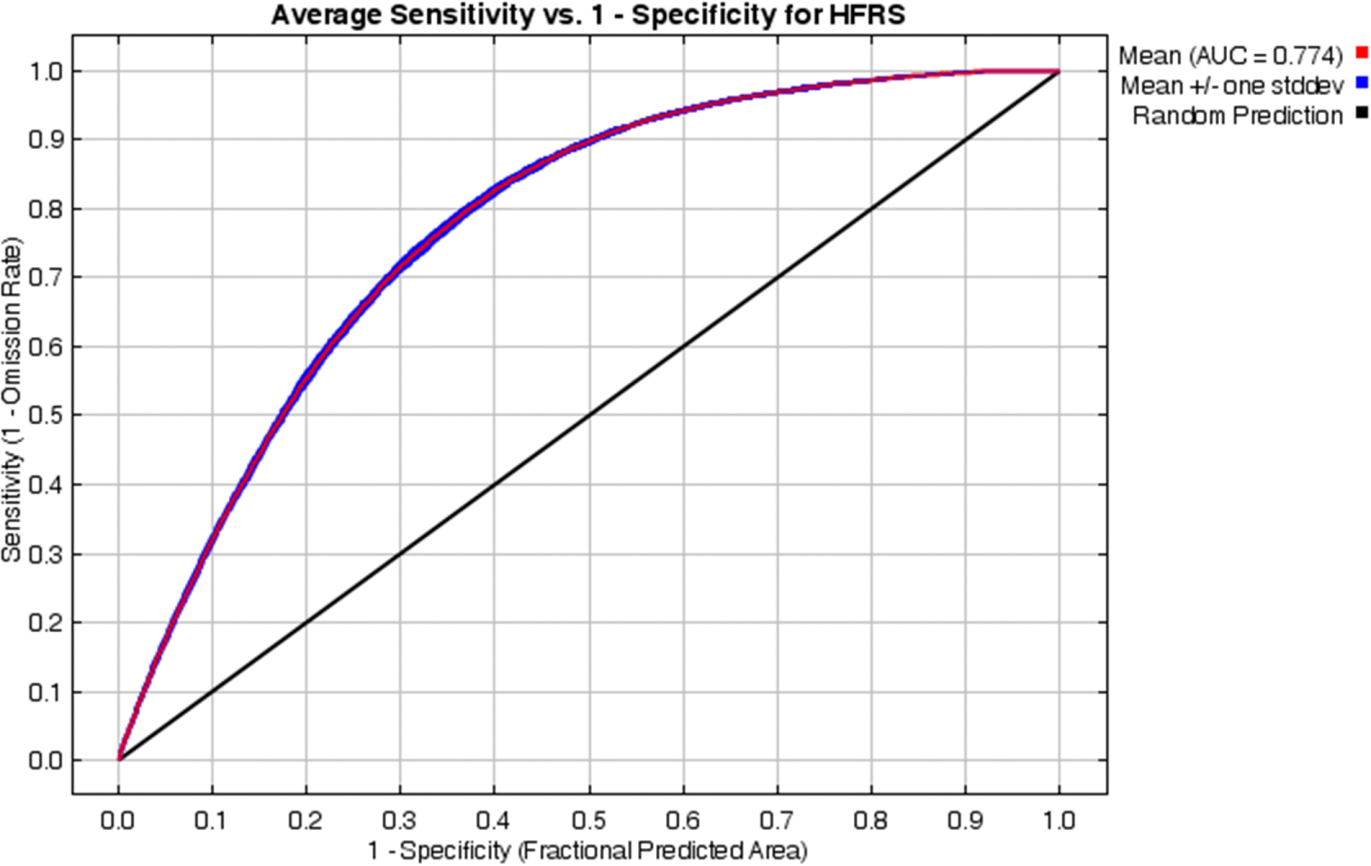
Receiver operating characteristic curve of the MaxEnt model.

By screening influencing variables, 6 variables were ultimately excluded, leaving 10 variables to be included in the model (Fig 3). By screening HFRS distribution data, 3,587 of the 4,837 distribution points were ultimately included in the model. By optimizing the model parameters, the final results showed that the modeling effect was optimal when FC was set to LQHPT and RM was set to 0.2 (S2 Fig). Additionally, the model’s AUC value was 0.77, indicating that the model is applicable (Fig 4).

**Fig 5:**
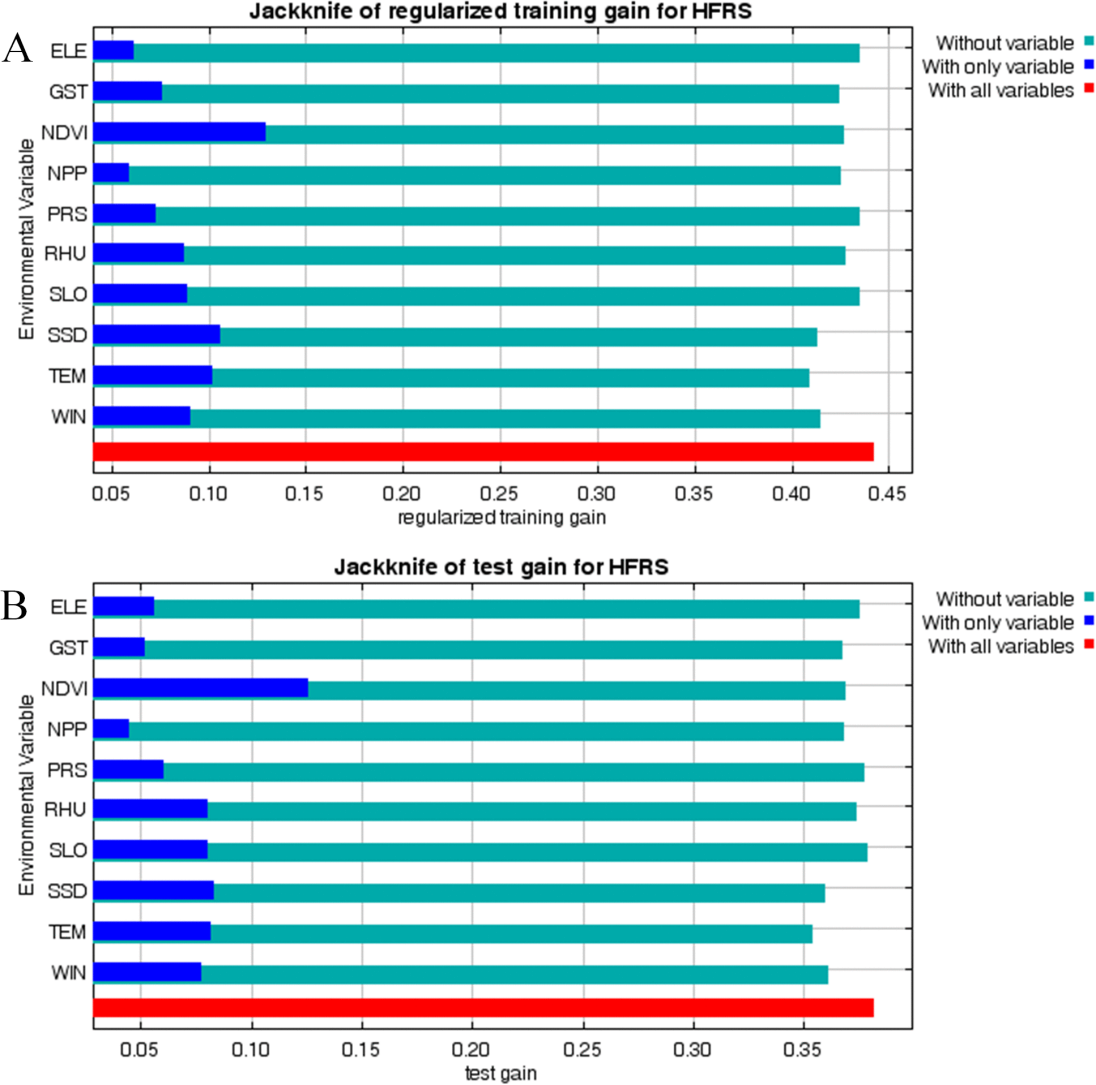
Result of the jackknife in MaxEnt model. **(A)** Jackknife of regularized training gain for HFRS; **(B)** Jackknife of test gain for HFRS.

In terms of contribution to the model, yearly average air temperature (TEM, 19.9%), normalized difference vegetation index (NDVI, 19.2%), yearly average relative humidity (RHU, 12.2%) and yearly average sunshine duration (SSD, 11.2%) ranked at the top, indicating that they are the main factors influencing the occurrence of HFRS (Table 1). Additionally, the jackknife test showed that TEM, NDVI, RHU, and SSD all fit the test and training data well, indicating that they capture the most effective information not included in the other variables (Fig 5).

**Fig 6:**
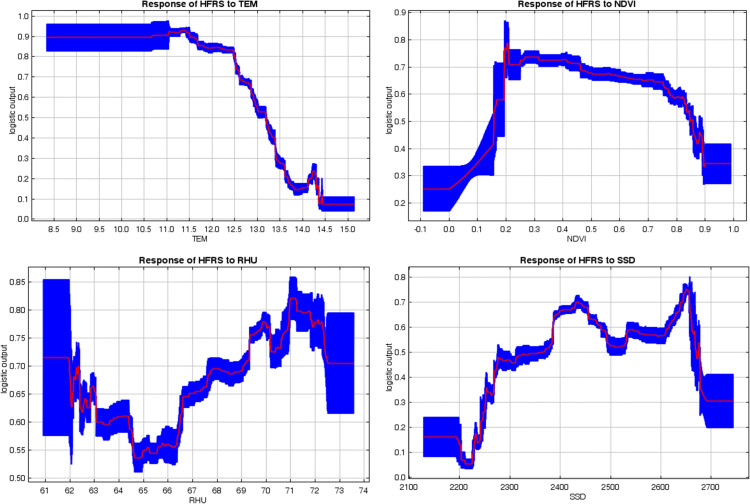
Response curves of HFRS to four significant factors in MaxEnt model.

Further response curve analysis revealed a nonlinear correlation between the probability of HFRS occurrence and each variable. As TEM increase, the probability of HFRS occurrence initially remained stable before declining sharply. The optimal TEM for HFRS occurrence was below 13.19°C. The response curve for NDVI exhibited an inverted “U” shape, with the most likely range for HFRS occurrence being 0.16–0.85. The response curve for RHU exhibited an “U” shape, with the most likely range for HFRS occurrence being 60.92%–73.56%. The response curve for SSD exhibited an inverted “U” shape, with the most likely range for HFRS occurrence being 2369.12h–2669.85h (Fig 6).

**Fig 7:**
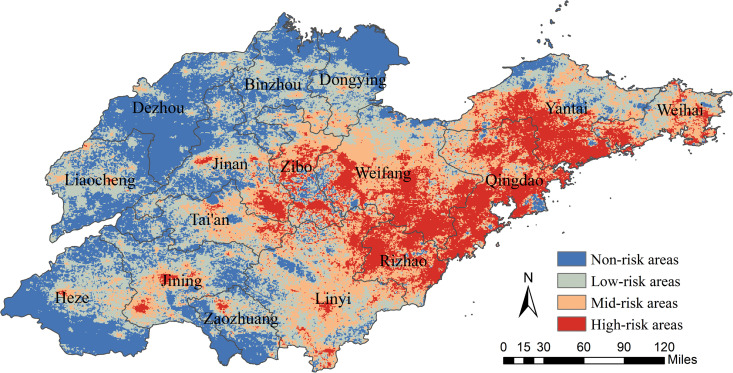
Predicted risk map of HFRS in Shandong Province, China. The base map is from the National Platform for Common GeoSpatial Information Services (https://cloudcenter.tianditu.gov.cn/administrativeDivision).

A risk map for HFRS occurrence in Shandong Province was also fitted using the MaxEnt model. The results showed that the areas of high-risk areas, mid-risk areas, low-risk areas, and non-risk areas were 26682.92 km^2^, 37557.63 km^2^, 47404.64 km^2^ and 46254.82 km^2^, respectively, accounting for 16.90%, 23.79%, 30.02%, and 29.29% of the total area of the province. High-risk areas for HFRS were primarily concentrated in the eastern and central regions of Shandong Province, while non-risk areas were mainly located in the western and northern regions. Some cities exhibited a distinct high-risk trend, including Qingdao, Zibo, Yantai, Weifang, and Rizhao (Fig 7).

## Discussion

Shandong Province in China is a traditional endemic area for HFRS. Since the first case was identified, Shandong has maintained over 50 years of HFRS prevention and control efforts. In particular, with the implementation of comprehensive intervention measures such as rodent control, vaccination, environmental improvement, and health education, great achievements had been obtained in HFRS prevention and control in Shandong Province [25]. However, in recent years, with the acceleration of urbanization, HFRS epidemics in some regions of China have shown significant fluctuations, and the current epidemic situation still faces many uncertainties [26]. Based on this, this study systematically revealed the recent distribution pattern of HFRS in Shandong Province by descriptive analysis and spatiotemporal scan analysis, and employed an optimized MaxEnt model to precisely explore the environmental factors influencing HFRS occurrence and identify future hotspot areas, thereby providing a basis for scientific prevention and control of HFRS.

This study showed that the incidence of HFRS in Shandong Province exhibited an overall fluctuating downward trend from 2018 to 2024. While enhanced public health interventions may be the primary cause, natural dynamics in the host animal population must also be considered. Studies from other epidemic areas in China confirmed that HFRS case fluctuations are closely linked to rodent density [27,28]. Therefore, this downward trend likely resulted from the combined effects of effective control measures and natural decline in rodent populations. However, the incidence rate of HFRS in Shandong Province remains relatively high compared to other provinces such as Hubei and Anhui [29,30]. Additionally, the case fatality rate in Shandong Province is also relatively higher compared to provinces like Zhejiang and Jiangxi [13,31]. This suggests that HFRS remains a serious public health issue and should be given high priority for prevention and control continuously in Shandong Province. Similar to previous study results [32,33], HFRS cases primarily involve middle-aged and elderly individuals, males, and farmers, indicating that agricultural activities, exposure to the outdoors, and male occupational behaviors (such as farming and warehouse management) remain key risk factors. This suggests that these groups should be targeted as high-risk populations for enhanced prevention and control measures.A study conducted in the early 21st century revealed that prior to 2005, Shandong Province exhibited a distinct double-peak incidence pattern for HFRS, with one peak occurring each spring and another during the autumn and winter months [34]. However, this study found that from 2018 to 2024, Shandong Province experienced only one peak annually during the autumn and winter months (October to December), with the spring peak having largely disappeared. This shift may be related to changes in the habitat of *Rattus norvegicus*, which is more active during spring. Advancements in agricultural techniques, improved housing conditions, and enhanced environmental sanitation have increasingly rendered residential areas inhospitable for *Rattus norvegicus*. This suggests that Shandong Province should adjust its HFRS prevention and control measures in a timely manner, shifting the focus of rodent control efforts toward *Apodemus agrarius*, which thrives in wild habitats. This strategic shift could effectively curb outbreaks during the autumn and winter seasons.

The primary advantage of spatiotemporal scanning analysis when applied to infectious disease surveillance is its ability to simultaneously consider both temporal and spatial dimensions of clustering. Previous studies have utilized spatiotemporal scanning analysis to investigate the clustering of severe fever with thrombocytopenia syndrome and brucellosis in Shandong Province [35,36]. This study employed spatiotemporal scanning analysis to precisely identify the specific high-incidence periods in HFRS high-risk areas, thereby determining the source type of the epidemic in these regions. This study identified four spatiotemporal clustering zones, three of which are located in the eastern, central, and northern parts of Shandong Province, with clustering periods occurring between October and December when the *Apodemus agrarius* is most active. This suggests that these three clustering zones are likely to be *Apodemus agrarius* epidemic foci. The other cluster zone is located in the Jining and Heze areas of southwestern Shandong, with the clustering period occurring between March and April. This period falls between the end of the autumn-winter peak and the beginning of the spring peak of HFRS, indicating that the cluster area is likely to be a mixed epidemic area. Previous studies have also confirmed that the Jining region is a mixed epidemic area for HFRS, consistent with the results of this study [37]. This suggests that Shandong Province should develop tailored prevention and control measures based on local conditions, such as formulating rodent control and extermination plans for different rodent species according to HFRS epidemic source types, and reasonably scheduling HFRS vaccine administration times based on the distinct epidemiological characteristics of each region.

In this study, an optimized MaxEnt model was first applied to HFRS. By systematically identifying the feature combination multiplier and regularization multiplier, this model better captures the true relationship between disease distribution and environmental factors, thereby generating more robust and interpretable predictive results. Meteorological factors can influence rodent population dynamics and thereby alter human-rodent interactions, and are widely recognized as important determinants of HFRS incidence [38,39]. This study identified temperature as a critical factor affecting HFRS occurrence, with the likelihood of HFRS gradually decreasing as temperatures rise. Tian et al. demonstrated that temperature affects rodent survival rates and density, altering the frequency of outdoor contact between humans and viruses, thereby influencing disease transmission [40]. Cold weather can affect rodent food supplies, potentially increasing their reliance on human habitats and thereby raising the risk of human infection [5]. This study also found that relative humidity exhibits a nonlinear relationship with the occurrence of HFRS. Shandong Province transitions between the humid subtropical and humid continental zones, featuring dry, long, and cold winters [41]. Rodents in dry environments may congregate near human settlements where water and food sources were abundant, increasing opportunities for human-rodent contact [39]. Previous studies have confirmed that relative humidity not only governs viral survival in the environment by affecting its physiological properties, but also influences aerosol formation. Aerosols in high-humidity environments are particularly conducive to viral transmission to humans [42]. Additionally, this study found that the response curve between sunshine duration and HFRS occurrence exhibits an inverted U-shape, with both excessively low and excessively high sunshine levels being detrimental to HFRS occurrence. This finding differs from previous research results. Past studies have found that sunlight exposure either has a positive effect on HFRS or shows no association [43,44]. This discrepancy may stem from the unique natural geographical environments of different regions.

Meteorological factors have altered the transmissibility of the virus to some extent, while socioeconomic factors are prerequisites for the occurrence of HFRS [45]. This study found that normalized difference vegetation index is another key factor influencing HFRS incidence, with the highest likelihood of HFRS occurring when normalized difference vegetation index range from 0.16 to 0.85. Normalized difference vegetation index represents surface vegetation coverage and serves as a common indicator reflecting both natural environmental and human socioeconomic factors. Previous studies have found that when normalized difference vegetation index is too high, human activity intensity decreases, thereby reducing the likelihood of HFRS occurrence [45]. Some scholars also suggested that normalized difference vegetation index exhibits a pronounced lag effect on HFRS outbreaks, as vegetation growth required time before translating into rodent population expansion and heightened viral transmission [46]. Additionally, this study generated a risk prediction map for HFRS, showing that high-risk areas are primarily concentrated in eastern and central Shandong Province. This suggests that in these regions, measures such as rodent control programs, vaccination of high-risk populations, and environmental modifications should be prioritized. In non-endemic areas, mechanisms for monitoring imported cases should be established to prevent HFRS transmission caused by population mobility or habitat changes.

This study has some limitations. First, the case data were obtained from the CISDCP, which is a passive surveillance system, meaning that a small number of undetected cases may not have been included in the study. Second, due to data acquisition restrictions, this study did not include rodent density, viral carriage rates, or population vaccination coverage. These factors could help explain the interaction mechanisms between rodents, environmental factors, and HFRS cases. These topics warrant further investigation in the future.

## Conclusion

From 2018 to 2024, the incidence of HFRS in Shandong Province showed a fluctuating downward trend, with a distinct temporal, regional, and population clustering pattern. HFRS exhibited a seasonal single-peak distribution, with middle-aged and elderly individuals and farmers being the primary high-risk groups, and the eastern and central regions of Shandong Province being the main epidemic areas. The occurrence of HFRS was influenced by multiple environmental factors, with temperature, normalized difference vegetation index, relative humidity and sunshine duration being the most critical factors. This helps to better understand the mechanisms of HFRS transmission and provides practical geographic information support for public health authorities to develop control strategies. The modeling methods and results of this study can be applied to similar HFRS-endemic regions to implement targeted control measures.

## Supporting information

S1 Table Demographic characteristics of HFRS cases in Shandong Province from 2018 to 2024. (DOCX)

S2 Table Spatiotemporal clusters of HFRS cases in Shandong Province at the county level, 2018–2024. (DOCX)

S1 Fig Map of Shandong Province in China. The base map is from the National Platform for Common GeoSpatial Information Services (https://cloudcenter.tianditu.gov.cn/administrativeDivision).
(TIF)

S2 Fig AICc value of parameter combinations based on the ENMeval calculation. (TIF)
