# Supplementary material for: Spatiotemporal pattern of hemorrhagic fever with renal syndrome and driving factors in Shandong Province of China, 2018–2024
Source: PLoS Negl Trop Dis. 2026 Feb 24;20(2):e0014023. doi: 10.1371/journal.pntd.0014023 (PMC12948312; doi:10.1371/journal.pntd.0014023)
Supplement: S1 Table — (DOCX) [file pntd.0014023.s001.docx]

**S1 Table. Demographic characteristics of HFRS cases in Shandong Province from 2018 to 2024.**

| Category | | Number of cases | Proportion (%) |
| --- | --- | --- | --- |
| Gender | male | 3541 | 73.21 |
|  | female | 1296 | 26.79 |
| Age | ≤19 | 107 | 2.21 |
|  | 20-39 | 805 | 16.64 |
|  | 40-59 | 2340 | 48.38 |
|  | ≥60 | 1585 | 32.77 |
| Occupation | Student | 90 | 1.86 |
|  | Teacher | 13 | 0.27 |
|  | Business service | 65 | 1.34 |
|  | Medical personnel | 8 | 0.17 |
|  | Workers | 309 | 6.39 |
|  | Farmers | 3829 | 79.16 |
|  | Clerical staff | 70 | 1.45 |
|  | Retired personnel | 113 | 2.34 |
|  | Household and unemployed | 258 | 5.33 |
|  | Unknown | 28 | 0.57 |
|  | Other | 54 | 1.12 |
