# Supplementary material for: Spatiotemporal pattern of hemorrhagic fever with renal syndrome and driving factors in Shandong Province of China, 2018–2024
Source: PLoS Negl Trop Dis. 2026 Feb 24;20(2):e0014023. doi: 10.1371/journal.pntd.0014023 (PMC12948312; doi:10.1371/journal.pntd.0014023)
Supplement: S2 Table — (DOCX) [file pntd.0014023.s002.docx]

**S2 Table. Spatiotemporal clusters of HFRS cases in Shandong Province at the county level, 2018–2024.**

| Variables | First cluster | Second cluster | Third cluster | Fourth cluster |
| --- | --- | --- | --- | --- |
| Radius (km) | 151.86 | 81.25 | 35.75 | 0 |
| Time frame | 2018/10 to 2018/11 | 2021/11 to 2021/12 | 2018/3 to 2018/4 | 2021/11 to 2021/12 |
| Population | 23040611 | 14425490 | 2591433 | 552583 |
| Number of counties | 29 | 18 | 4 | 1 |
| Cluster counties | Shinan, Shibei, Xihaian, Laoshan, Licang, Chengyang, Jimo, Jiaozhou, Pingdu, Laixi, Laiyang, Laizhou, Zhaoyuan, Qixia, Haiyang, Weicheng, Hanting, Fangzi, Kuiwen, Changle, Zhucheng, Anqiu, Gaomi, Changyi, Rushan, Donggang, Lanshan, Wulia, Ju | Licheng, Zhangqiu, Laiwu, Gangcheng, Zichuan, Zhangdian, Boshan, Zhoucun, Yiyuan, Linqu, Sishui, Taishan, Daiyue, Xintai, Yinan, Yishui | Yutai, Jinxiang, Chengwu, Juye | Shanghe |
| Annual cases/100,000 | 10.9 | 3.9 | 4.5 | 10.2 |
| Observed/expected | 16.22 | 5.79 | 6.64 | 15.17 |
| Relative risk | 17.68 | 5.89 | 6.67 | 15.19 |
| Loglikelihood ratio | 785.24 | 87.98 | 20.92 | 16.07 |
| P value | < 0.01 | < 0.01 | < 0.01 | < 0.01 |
