# Supplementary figures and images for: Spatiotemporal pattern of hemorrhagic fever with renal syndrome and driving factors in Shandong Province of China, 2018–2024
Source: PLoS Negl Trop Dis. 2026 Feb 24;20(2):e0014023. doi: 10.1371/journal.pntd.0014023 (PMC12948312; doi:10.1371/journal.pntd.0014023)

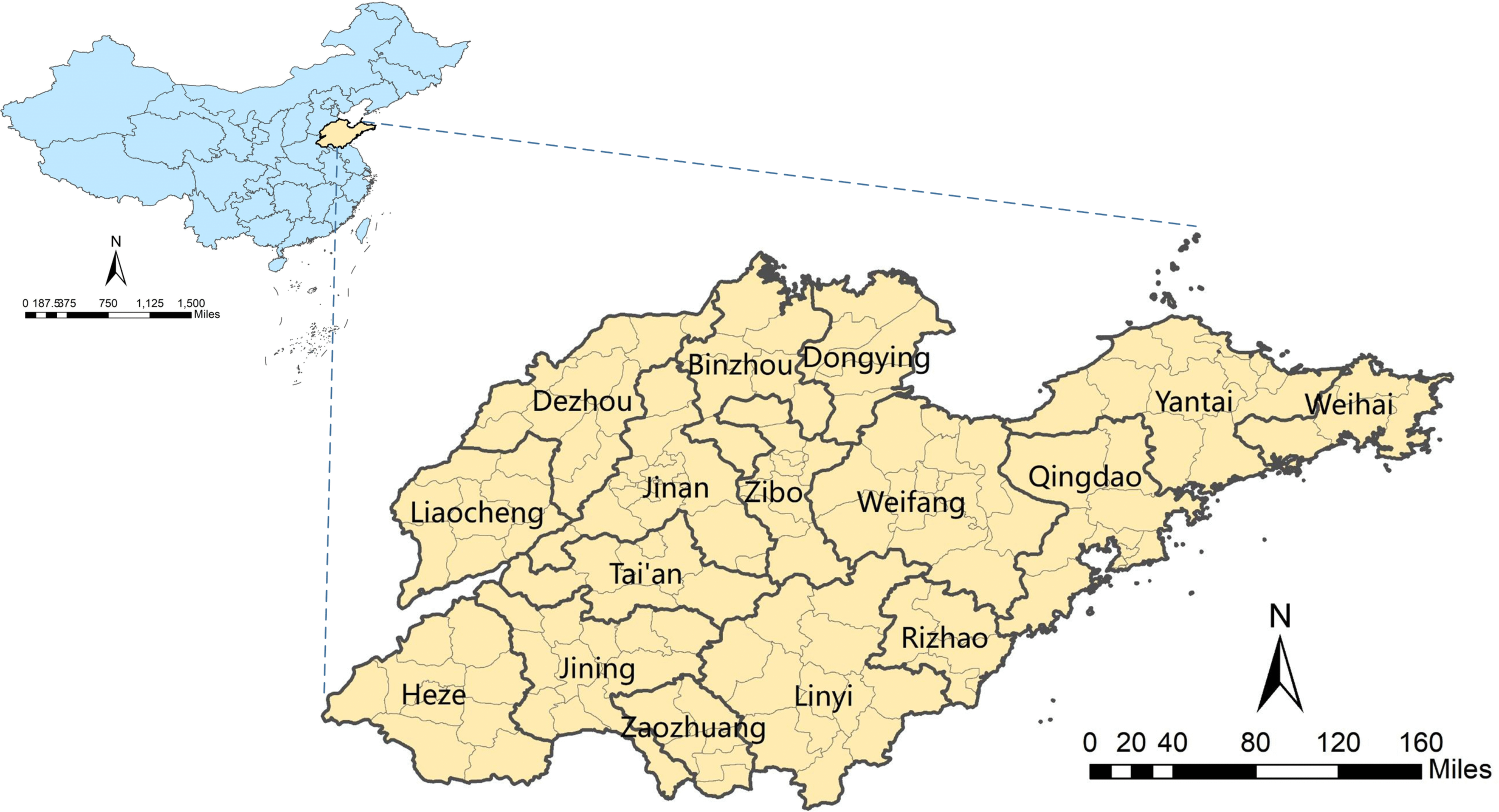

Supplement: S1 Fig — The base map is from the National Platform for Common GeoSpatial Information Services (https://cloudcenter.tianditu.gov.cn/administrativeDivision). (TIF) [file pntd.0014023.s003.tif]

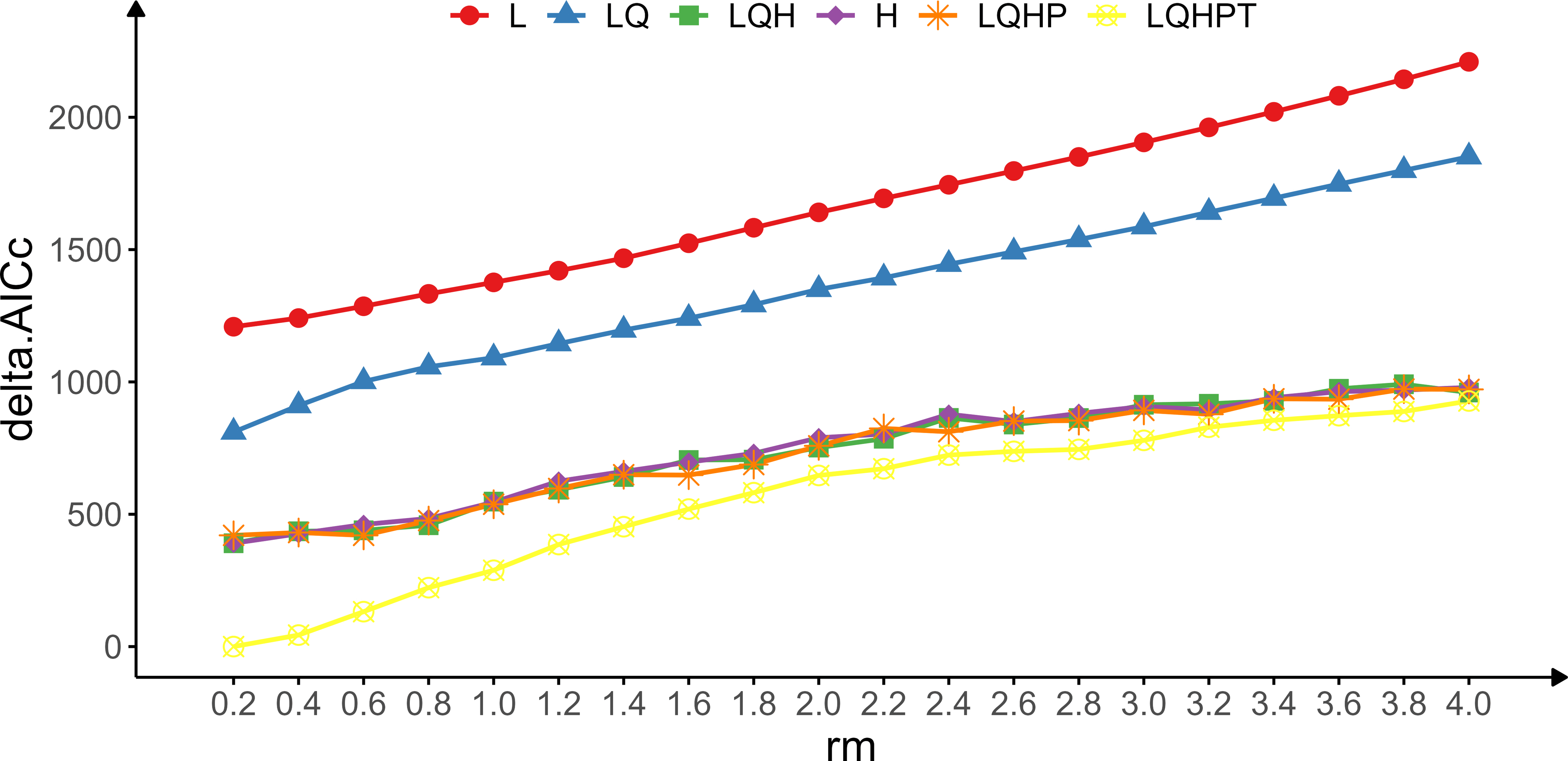

Supplement: S2 Fig — (TIF) [file pntd.0014023.s004.tif]
